# A Theoretical Study of Love Wave Sensors Based on ZnO–Glass Layered Structures for Application to Liquid Environments

**DOI:** 10.3390/bios6040059

**Published:** 2016-12-02

**Authors:** Cinzia Caliendo, Muhammad Hamidullah

**Affiliations:** Institute of Photonics and Nanotechnologies, IFN-CNR, Via Cineto Romano 42, 00156 Rome, Italy; m.hamidullah@ifn.cnr.it

**Keywords:** Love modes, viscosity, mass density, sensitivity, wave attenuation, wave velocity, ZnO, glass, biosensor, liquid environment, Rayleigh wave

## Abstract

The propagation of surface acoustic Love modes along ZnO/glass-based structures was modeled and analysed with the goal of designing a sensor able to detect changes in the environmental parameters, such as liquid viscosity changes and minute amounts of mass supported in the viscous liquid medium. Love mode propagation was modeled by numerically solving the system of coupled electro-mechanical field equations and Navier–Stokes equations. The phase and group velocities and the attenuation of the acoustic wave propagating along the 30° tilted c-axis ZnO/glass structure contacting a viscous non-conductive liquid were calculated for different ZnO guiding layer thicknesses, added mass thicknesses, and liquid viscosity and density. The three sensor responses, i.e., the wave phase and group velocity, and attenuation changes are calculated for different environmental parameters and related to the sensor velocity and attenuation sensitivities. The resulted sensitivities to liquid viscosity and added mass were optimized by adjusting the ZnO guiding layer thickness corresponding to a sensitivity peak. The present analysis is valuable for the manufacture and application of the ZnO-glass structure Love wave sensors for the detection of liquid properties, such as viscosity, density and mass anchored to the sensor surface.

## 1. Introduction

Love waves are acoustic modes that propagate along the surface of a structure comprising a layer on top of a half-space, when the shear bulk acoustic wave velocity of the layer is slower than that of the substrate. The difference between the mechanical properties of the layer and the substrate results in an entrapment of the acoustic energy in the guiding layer. The Love waves are shear horizontally polarized normally to the wave propagation direction. The shear polarization and the guiding layer effect make the Love wave sensors suitable for liquid application and very sensitive to any changes occurring on their surface, such as those related to mass loading and changes of the liquid viscosity and density [1,2,3,4,5]. At the guiding layer surface, the liquid is sheared and shear waves in the liquid are set up. Due to the attenuation of shear waves in viscous liquids, the energy is dissipated rapidly and the shear waves do not penetrate far into the liquid. The Love mode velocity is reduced and the attenuation is increased along its propagation path due to energy lost in shearing the fluid: the resulting velocity change and the attenuation depend on the viscosity of the fluid as well as on the guiding layer thickness.

Piezoelectric wurtzite ZnO thin film technology has been widely used for many years for the fabrication of surface acoustic wave (SAW) devices onto non piezoelectric substrates, such as silicon, glass, and sapphire, to name just a few. When the hexagonal ZnO film has its c-axis orthogonal or parallel to the substrate free surface, it is effective in the transduction of Rayleigh waves or Love waves: the electric field is coupled to Rayleigh waves in the former case, and to Love waves in the latter. When the piezoelectric film has its c-axis tilted at an angle μ with respect to the normal to the substrate surface, for wave propagation along the <100> direction, two types of surface modes propagate: the Love-like, with predominant in-plane shear horizontal polarization, and the Rayleigh-like, with a prevailing sagittal polarization. Both the two modes are coupled to the electric field via the effective piezoelectric constants of the film. Thus, Love wave devices can be directly fabricated on silicon or glass substrate by using the thin piezoelectric film technology whose characteristics depend on the piezoelectric guiding layer thickness and on the c-axis tilt angle μ. Depending on the material’s crystallographic orientation, both the in-plane polarized Love mode and the out-of-plane polarized surface acoustic waves (SAWs) can be excited on the same multilayered structure. The Love mode and the SAW play two different roles in the same sensing platform: the former is suitable for liquid environment characterization, while the latter is suitable for mixing and pumping small liquid volumes. Matatagui et al. reported the development of a Love wave immunosensor with microfluidic technology to detect potentially pathogenic microorganisms in real time [6]. Most Love mode sensors are implemented on quartz covered by a SiO_2_ guiding layer, but Love mode sensors implemented on silicon or glass substrate materials offer the great advantage of the sensors integration with the surrounding electronic circuits [7]. 

In this paper, we theoretically investigate the performance of a glass/ZnO Love wave sensor which is suitable for the fabrication of a biological sensing platform including Rayleigh wave-based microfluidic devices. The organization of the present article begins with a study of the mass sensitivity of a two-layer model, a lossless glass substrate covered by a 30° tilted c-axis ZnO guiding layer, in vacuum. Then, a viscous Newtonian liquid is introduced that contacts the guiding layer free surface, and a complex wave velocity is defined for the three-layer system (substrate, guiding layer, liquid); the velocity and attenuation sensitivities to viscosity and to an added mass are then calculated. Finally, the capability of the Love wave sensor is studied for gravimetric detection in a viscous liquid environment. The selective detection of chemical and biochemical species in liquid is simulated by applying an interface layer onto the guiding layer surface, and the mass sensitivity of a four-layer model (substrate, guiding layer, interface layer, liquid) was then defined for different viscosity values. The effect of the sensitivity dispersion of Love wave sensor is investigated, and we focused on the guiding layer thickness optimizing the mass sensitivity for the first Love wave mode. The aim of the present theoretical calculations is to investigate the influence of the ZnO layer thickness on the advances made on the sensitivity of a Love wave device for biosensing applications. 

## 2. Love Wave Sensors in ZnO–Glass Layered Structures for Application to Liquid Environments: Statement of the Problem and Basic Equations

**Figure 1:**
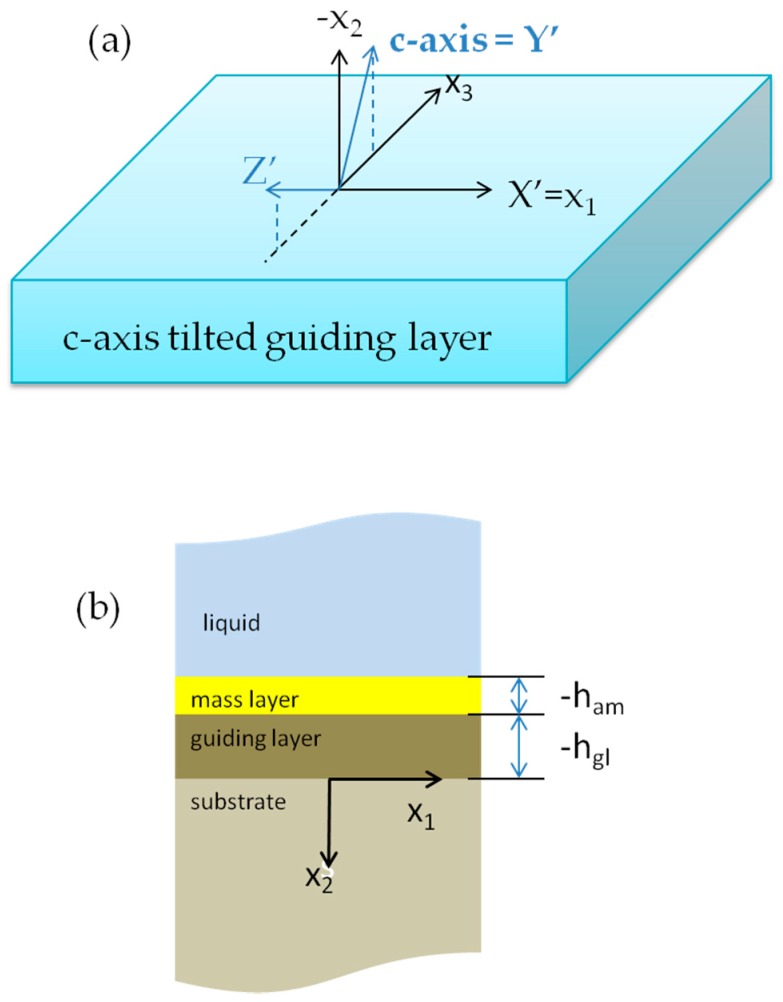
(**a**) The laboratory coordinates system x_1_ x_2_ x_3_ and the ZnO layer coordinate system X’ Y’ Z’; (**b**) the geometry of the substrate/guiding layer/added mass layer/liquid system.

The 30° tilted ZnO coordinate system and the ZnO/glass layered structure here studied are illustrated in Figure 1a,b. The substrate is assumed to be an isotropic dielectric elastic medium, glass, that occupies the half-space x_2_ > 0; the guiding layer is assumed to be a ZnO film with the c-axis tilted 30° with respect to the surface normal; the added mass layer, deposited onto the guiding layer, is in contact with the viscous liquid half-space. The x_2_ = 0 plane is the interface between the substrate and guiding layer, the x_2_ = −h_gl_ and x_2_ = −h_am_ planes are the guiding layer-added mass and added mass-liquid interfaces. H = h_gl_ + h_am_, ρ_l_ and η are the mass density and viscosity of the viscous liquid. The Love wave is assumed to propagate along the x_1_ direction and to be shear horizontally polarized. U_3_ is the only non-zero particle displacement component; no variation of U_3_ in the x_3_ direction is assumed.

The dynamic equations for the three studied systems, the substrate/guiding layer, the substrate/guiding layer/viscous liquid, and the substrate/guiding layer/added mass layer/viscous liquid half-space are described in the Supplementary Materials. Solutions to the wave equations are sought which decay to zero with depth below the surface x_2_ = 0 and in the liquid half-space; inside the guiding and added mass layers, the displacement varies sinusoidally. When these solutions are substituted into the electrical and mechanical continuity and boundary conditions, a set of algebraic equations is obtained. In order to have a non-trivial solution to this set of equations, the determinant of the coefficients must vanish. For a specific wavelength *λ* and layers thickness, successive values of wave velocity are chosen that lay between the shear bulk wave velocity of the substrate and of the guiding layer, until the boundary conditions determinant is made as close to zero as desired. For the glass/ZnO layer system, real velocity values are obtained under the assumption of lossless materials and in vacuum. For the half-space/ZnO/viscous liquid system, the wave vector becomes a complex number, *k* = *k*_0_ + *j·*α, where *k*_0_ = 2·π/λ, α is the wave attenuation. When the boundary condition determinant is set equal to zero, a complex dispersion equation is obtained; the system of two equations, the real and imaginary parts of the dispersion equation, was numerically solved by using the Levenberg–Marquardt–Fletcher method implemented within a Matlab routine, and the real and imaginary parts of the Love wave velocity were finally calculated, $v^{im}$ and $v^{real}$. From $v^{im}$ and $v^{real}$ the phase and group velocities, $v_{ph}$ and $v_{gr}$, and the attenuation α of the Love waves are then calculated for three different ZnO/glass-based structures: (1) substrate-guiding layer-vacuum; (2) substrate-guiding layer-liquid; and (3) substrate-guiding layer-added mass-liquid. The Love wave phase and group velocity and attenuation sensitivities to the mass density and viscosity of the liquid environment were calculated, as well as the sensitivity to a mass anchored onto the device surface while contacting a viscous liquid.

## 3. Mass Sensitivity of the Vacuum–ZnO–Glass Structure

**Figure 2:**
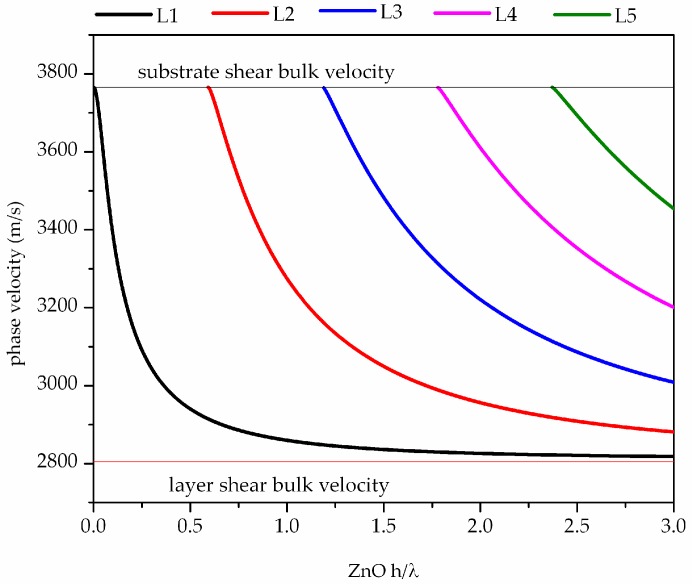
The phase velocity dispersion curves of the first five Love modes propagating along the 30° tilted ZnO/glass/vacuum structure.

The system of coupled electro-mechanical field equations for the substrate and piezoelectric guiding layer were solved numerically under the hypothesis that the ZnO layer is piezoelectric and has the c-axis 30° tilted, and the glass substrate is isotropic, as described in the Supplementary Materials. The substrate and layer are assumed to be lossless and their material parameters (elastic, dielectric, piezoelectric constants and mass density) are deduced from reference [8]. The elastic, piezoelectric and dielectric constants of the ZnO were rotated according to the Bond matrix method described in reference [9]. The phase velocity dispersion curves of the first five Love modes propagating along the ZnO/glass substrate were calculated for both the open and short circuited guiding layer surfaces. The calculated phase velocity dispersion curves are shown in Figure 2.

**Figure 3:**
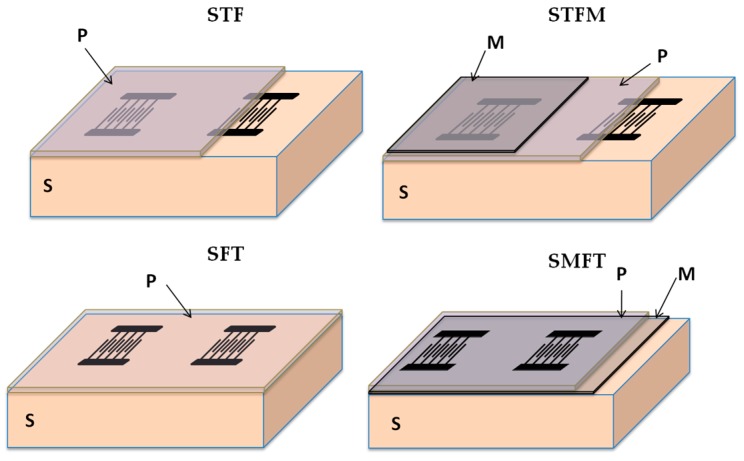
The four coupling configurations for the layer/substrate system.

As for the SAWs, Love waves are excited and detected by means of interdigital transducers (IDTs). In designing a Love wave device, an important feature to be obtained is a low insertion loss, which can be achieved by selecting a material with a large electromechanical coupling coefficient, *K*^2^. The value of *K*^2^ is directly related to the IDTs electrical-to-mechanical energy conversion efficiency; hence, it determines the radiation resistance of the IDTs that are fabricated on the piezoelectric guiding layer surface. The layer/substrate combination allows the implementation of four different coupling configurations, with the IDTs placed on one of the ZnO layer surfaces, with or without a floating electrode on the opposite surface. The substrate/film/transducer (SFT) configuration refers to a coupling structure with the IDTs positioned on the ZnO free surface; when a floating metallic plane (M, metal) is placed at the ZnO/glass interface, the configuration is called SMFT. The substrate/transducer/film (STF) configuration refers to a coupling structure with the IDTs positioned at the ZnO/glass interface; when the floating metallic plane is positioned at the ZnO free surface, the configuration is called STFM. The *K*^2^ can be obtained by calculating the perturbation of the wave velocity when the tangential electric field component is shorted out at the ZnO surface: $K^{2}=2\times \left(v_{free}-v_{met}\right)/v_{free}$, where *v_free_* and *v_met_* are the phase velocities at the electrically opened and shorted surfaces of the ZnO film. The *v_met_* is obtained by the insertion of a perfectly conductive and infinitesimally thin film at the interfaces where the IDTs and the floating plane are located in each of the four coupling structures. The mechanical effect of the IDT and floating electrode was ignored as they were assumed to be infinitely thin. Figure 3 shows the four coupling configurations for the layer/substrate structure, where P, S, and M stand for piezoelectric layer, substrate and metal plane, respectively.

By denoting as *v_ij_* (for *i, j* = m, f) the wave velocity referred to the electrical boundary conditions at the lower (first index, *i*) and upper (second index, *j*) layer surface, the following approximated equations were used to calculate the coupling constant of the four structures:

(1) KSFT2=2⋅[(vff−vfm)vff]

(2) KSTF2=2⋅[(vff−vmf)vff]

(3) KSMFT2=2⋅[(vmf−vmm)vmf]

(4) KSTFM2=2⋅[(vfm−vmm)vfm]

**Figure 4:**
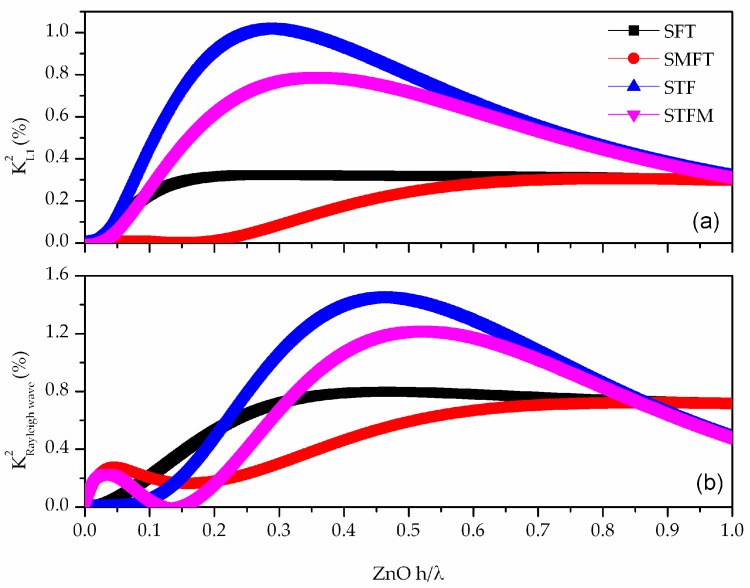
The *K*^2^ vs. ZnO normalized thickness, h/λ, of the first Love mode (**a**) and Rayleigh wave (**b**), for the four coupling configurations.

The $K^{2}$ of the first five Love modes was calculated for different guiding layer thicknesses and, as an example, Figure 4a shows the *K*^2^ dispersion curves of the first Love mode L_1_, for the four coupling configurations. As can be seen from Figure 4a, L_1_ mode propagating along the dispersive structure is highly sensitive to the electrical boundary conditions.

The STF configuration, as well as the STFM, is quite efficient, and offers the advantage of separating the IDTs from the environment; this feature is particularly useful in liquid sensing as well as in microfluidic applications. Figure 4b shows the *K*^2^ vs. ZnO normalized thickness of the Rayleigh wave that propagates along the same layer/substrate structure, for the four coupling configurations. The Rayleigh wave travels along the surface of the propagating medium and the most of its energy is concentrated in one wavelength depth from the surface; it is elliptically polarized (U_1_ and U_3_ are non-zero, U_2_ ≈ 0) and electrically coupled as well as the Love wave. For ZnO h/λ = 0.3, both the two waves show similar and quite good *K*^2^ (~1%) for the STF configuration. In presence of a liquid phase contacting the device surface, the two waves travelling along the same lab-on-chip substrate play different roles. The Love mode is suitable for liquids characterization, while the elliptically polarized Rayleigh wave is suitable for micro-fluidic applications.

The mass sensitivity *S_m_* in vacuum of the first five Love modes was calculated as the relative phase velocity shift per unit added mass

(5) Sm·λ=((vmass− vfree)vfree)/(ρmass·hmass)

where *ρ_mass_* and *h_mass_* are the added mass density and thickness, and *v_mass_* is the wave velocity perturbed by the mass loading effect. The additional mass was modeled as a very thin lossless layer of material contacting the ZnO layer surface. The mass loading effect dominates over the elastic effect of the added layer since the velocity of this perturbing layer is much less than that of the material supporting it. Thus, the added mass layer does not perturb the acoustic field profile inside the guiding layer. The calculated sensitivity is negative since the mass loading effect decreases the wave velocity. The mass sensitivities of the first five modes propagating along the glass/ZnO STF structure, as well as for the Rayleigh wave, were calculated at the ZnO h/λ value corresponding to the peak of the derivative of the phase velocity with respect to h/λ. The S_m_·λ values obtained are listed in Table 1 where it can be seen that the peak sensitivity decreases with increasing the mode order. The highest sensitivity corresponds to the L_1_ mode: supposing that λ = 10 μm, an L_1_ sensitivity equal to −55.7 m^2^/kg is expected that corresponds to a ZnO thickness equal to 0.47 μm. 

## 4. Viscosity Sensitivity of the Liquid–ZnO–Glass Structure

**Figure 5:**
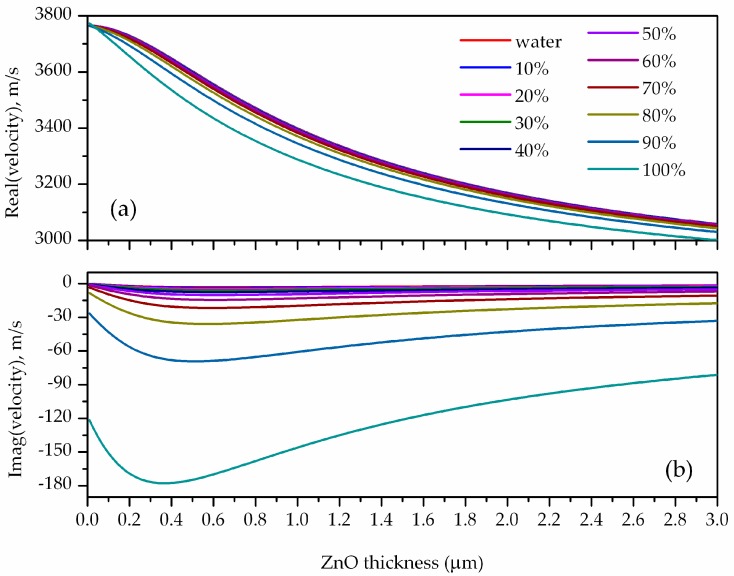
The real (**a**) and imaginary part (**b**) of the L_1_ mode phase velocity vs. the guiding layer thickness for different water/glycerol mixtures.

A simplified model, described in the Supplementary Materials, was used to numerically solve the four boundary equations system for the substrate/guiding layer/viscous (Newtonian) non-conductive liquid system: the ZnO layer was assumed isotropic and its elastic constants were made equal to the stiffened elastic constants of the 30° tilted piezoelectric counterpart. The viscous liquid was supposed to be a mixture of water and glycerol; the fraction of glycerol by volume ranged from 0 (only water) to 1 (only glycerol) and the $\sqrt{\rho_{l}\cdot \eta }$ ranged from 1 to 42.27 kg·m^−2^·s^0.5^ [10]. The system of the mechanical field equations and Navier–Stokes equations have been numerically solved and an analytical expression for the complex dispersion equation of Love waves has been established, as described in the Supplementary Materials. The two-equation system, the real and imaginary parts of the dispersion equation, was numerically solved by the Levenberg–Marquardt–Fletcher method in a Matlab routine. The effects of both the viscosity η and mass density *ρ_l_* of a water/glycerol mixture on the wave velocity and attenuation was analyzed numerically. The real and imaginary parts of the phase velocity of the L_1_ mode were calculated for different guiding layer thicknesses and for different water/glycerol mixtures. Figure 5a,b show the real and imaginary parts of the L_1_ mode velocity, v_r_ and v_i_, versus the guiding layer thickness for different water/glycerol mixtures and supposing λ = 10 μm.

**Figure 6:**
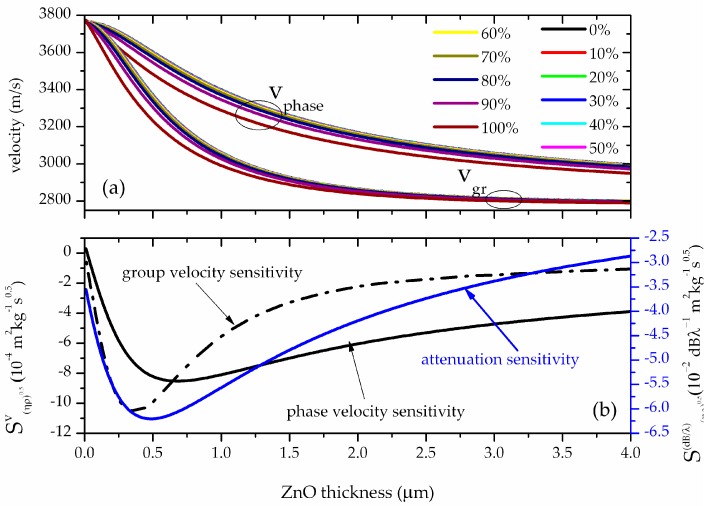
(**a**) The phase and group velocity dispersion curves for different water/glycerol percentage values; (**b**) the phase and group velocity, and the propagation loss sensitivities to $\sqrt{\rho \cdot \eta }$ vs. the ZnO thickness, for λ = 10 μm.

The calculated data clearly show that while the percentage of glycerol in water increases, the wave velocity decreases in respect to the velocity along the bare substrate in pure water and the attenuation increases. The group velocity *v_gr_* versus the ZnO layer thickness was calculated, for different water/glycerol mixtures, by applying the equation $v_{gr}=v_{ph}\left[1+\frac{h}{v_{ph}}\frac{\partial v_{ph}}{\partial h}\right]$. Figure 6a shows the group velocity dispersion curves for different water/glycerol mixtures. The relative velocity shift (vg/w−vwater)vwater and the propagation loss α = 2·π·log(e)·v_i_/v_r_ of the L_1_ mode vs. $\sqrt{\rho_{l}\cdot \eta }$ were calculated for different ZnO thicknesses (from 0 to 4 μm), with *v^water^* and *v^g/w^* being the wave velocity in pure water and in glycerol/water mixture. The attenuation and velocity relative changes vs. $\sqrt{\rho_{l}\cdot \eta }$ were linearly fitted, for each ZnO thickness value, and the slopes, i.e., the phase and group velocity and attenuation sensitivities Sρ·ηvph=(Δvphvhpwater)/ρl·η, Sρl·ηvgr=(Δvgrvgrwater)/ρl·η and $S_{\sqrt{\rho \cdot \eta }}^{att}$ = α/$\sqrt{\rho_{l}\cdot \eta }$, were plotted vs. the ZnO thickness; the calculated sensitivities dispersion curves are shown in Figure 6b. 

As it can be seen in Figure 6b, the three sensitivities increase with increasing ZnO layer thickness and reach a peak after which, with increasing guiding layer thickness, they decrease, thus confirming that the thickness of the guiding layer is a crucial parameter that can be varied to achieve a more sensitive device. From comparing the phase and group velocity sensitivities in Figure 6b, it can be noted that the latter may be larger than the former, and its peak can also be sharper [11]. Both the group and phase velocity can represent a sensor response, other than the attenuation. The phase velocity can be experimentally estimated by measuring the operating frequency *f = v_ph_/λ* of the sensing device at the minimum insertion loss of the scattering parameter S_12_. The group velocity can be estimated by measuring the group time delay τ* = L/v_gr_* of the sensing device at the minimum insertion loss of the scattering parameter S_12_ in the time domain, being L the acoustic wave delay path.

The phase velocity peak sensitivity corresponds to a ZnO layer approx. 0.46 μm thick. According to the calculations described in the previous paragraph, a Rayleigh wave propagates simultaneously in 30° tilted ZnO/glass/vacuum: the surface wave travels at 2643 m/s with *K*^2^ = 1.44% for the STF configuration. When the device surface contacts the liquid phase, part of the SAW refracts into the liquid as a longitudinal wave and the mode changes to a leaky SAW. The refracted wave moves along the direction given by the Rayleigh refraction angle $\theta_{Rayleigh}=\arcsin\left(\frac{v_{liq}}{v_{device}}\right)$*,* where *v_device_* and *v_liq_* represent the acoustic wave velocities of the device and the fluid, respectively. For a SAW propagating on 30° tilted ZnO/glass (with h/λ = 0.3) while contacting a pure water or pure glycerol environment, the resulting Rayleigh angles are about 34° and 46°, respectively. The refracted longitudinal waves generate a force in their propagation direction and induce flow within the confined liquid. The boundaries of the liquid drop reflect the actuated liquid and lead to internal streaming. For both the STF and STFM configurations there exists a range of ZnO thickness corresponding to quite good *K*^2^ for the Rayleigh wave and the first Love mode. The unmetallized Love mode propagation path of the STF configuration makes this device suitable for detecting both the liquid conductivity and viscosity, while the STFM configuration, due to the presence of the floating metal electrode that shortcircuits the wave electrical potential, is insensitive to the liquid conductivity. Thus, Rayleigh wave-based STF and L_1_-based STF and STFM configurations can be integrated on the same ZnO/glass substrate to implement a system able to sense mass, viscosity and conductivity changes of the solid-liquid interface.

## 5. Mass Sensitivity of the Liquid–ZnO–Glass Structure

**Figure 7:**
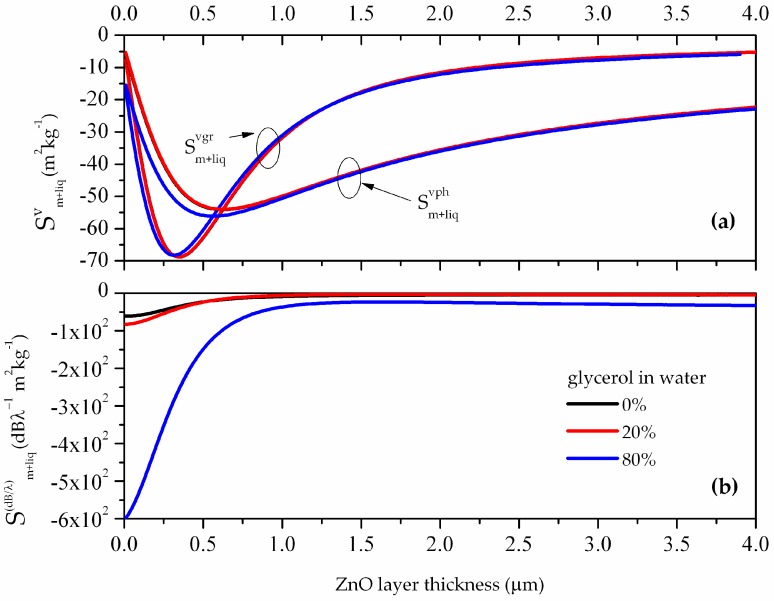
(**a**) The phase and group velocity sensitivities and (**b**) the attenuation sensitivity to added mass in liquid vs. the guiding layer thickness, for different liquid environment viscosities.

A system of six boundary equations for the four-layer structure (substrate, guiding layer, added mass layer, viscous liquid half-space) was numerically solved under the hypothesis that the ZnO layer is isotropic, as in the previous paragraph, and an analytical expression for the complex dispersion equation of Love waves was established, as described in the Supplementary Materials. The wave phase and group velocity and the attenuation perturbed by the added-mass were calculated for different liquid viscosity values and for different thicknesses of the guiding layer, in order to simulate the performances of a gravimetric sensor operating in liquid. The obtained results show that the imaginary part of the phase velocity is highly affected by the liquid viscosity while the real part changes very little. Thus in this case, the attenuation represents a valid sensor response, especially in the presence of liquids as viscous as 80% of glycerol in water. The gravimetric sensitivities of the phase and group velocity, and of the attenuation, $S_{m+liq}^{v}$ and S(dBλ), were calculated for different guiding layer thicknesses and liquid environment viscosity values, for λ= 10 μm; the calculated data are shown in Figure 7a,b.

As can be seen in Figure 7a,b, the phase velocity is far less sensible to added mass in viscous liquid than the attenuation. The maximum phase and group velocity gravimetric sensitivities are about −55 and −68 m^2^/kg at h ≈ 0.6 and 0.35 μm, respectively. The attenuation sensitivity for the three viscosities has the same behavior: it decreases with increasing the ZnO layer thickness and reaches a plateau at about 1μm ZnO thickness. In the ZnO thickness range from 0.35 to 0.6 μm, at 80% of glycerol in water, the attenuation sensitivity ranges from −300 to −100 dB/λ per unit mass.

## 6. Mass Sensitivity of the Liquid-Sensing Layer–ZnO–Glass Structure

**Figure 8:**
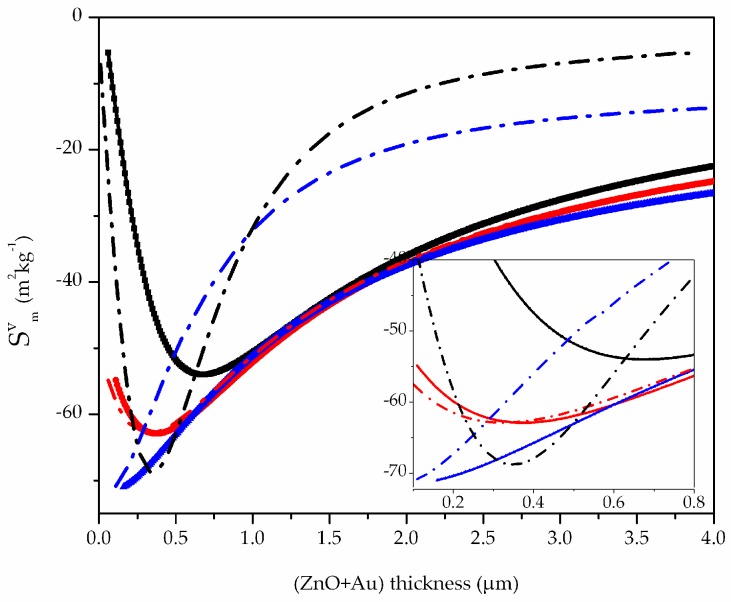
The mass sensitivities of the phase (solid lines) and group velocity (dotted lines) of the Love mode propagating along: the glass/ZnO/water, the black curves; glass/ZnO/Au (50 nm thick)/water, the red curves; glass/ZnO/Au (100 nm thick)/water, the blue curves. The abscissa represents the total thickness of the ZnO and Au layers.

The glass/ZnO sensor surface is supposed to be covered by a sensing Au layer able to absorb a thin mass layer while contacting the viscous liquid environment; this structure simulates the changes in the properties of the propagating acoustic wave caused by a biochemical interaction that takes place at the sensing area and causes a sensor response due to the mass loading effect. This can be the case for an indirect immunosensor that uses a thin gold layer as a label that immobilizes the recognition element at its surface via direct absorption mechanism. The mass sensitivities of the wave velocity and attenuation loss were estimated by following the calculation method outlined in the previous paragraph and fixing the thickness of the Au layer that covers the ZnO. Two Au layer thickness values were considered, 50 and 100 nm. Then the mode velocity and attenuation perturbed by increasing the Au sensing layer thickness were calculated in order to estimate the velocity and attenuation gravimetric sensitivities. In this case, the Au layer behaves as an interactive material that favorites the binding of an added mass in liquid environment. The phase and group velocity and attenuation sensitivities to the added mass in water were calculated for different Au layer thicknesses, and the results are shown in Figure 8. As it can be seen, the group (dotted lines) and phase velocity sensitivities (solid lines) are affected by the thickness of the sensing layer: for 0 thickness Au layer (black curves), the former is more sensitive than the latter; for 50 nm thick Au layer (red curves), the two sensitivities are comparable; for an Au layer 100 nm thick (blue curves), the phase velocity is more sensitive than the group velocity.

## 7. Discussion

There is a need for highly sensitive, simple and low-cost sensors for the detection of lifestyle-related diseases, pregnancy tests, and disease antigens in liquid samples such as serum, urine, or saliva, to cite just a few. Love wave sensors represent a valid answer to this need. Although it has been widely demonstrated that the Love wave sensor is able to sense the liquid properties (such as density and viscosity), the main objective for the biosensor is to detect the mass variations of a sensitive layer (i.e., the bio-film) that covers the surface of the sensor; the bio-film is able to adsorb specific genes or antigenes and consequently increases its mass. ZnO films with the c-axis tilted at an angle μ with respect to the normal of the device surface are useful for integrated bio-sensing and microfluidic applications as they are effective in the Love mode as well as SAW transduction. Depending on the guiding layer thickness and electrical boundary conditions, the acoustic waves generated along the ZnO–glass device can also induce acoustic streaming of the fluid to be characterized. 

There is a wide experimental evidence of the growth of 30° tilted ZnO films with the rf sputtering technique [12,13,14] or by PLD [15]. The 30° tilt angle represents a tradeoff between high structural quality of the currently grown films and quite good *K*^2^ of both the first Love mode and the Rayleigh wave. Both the Rayleigh wave and Love mode travelling along the 30° tilted ZnO–glass structure exhibit a *K*^2^ that is highly affected by the electrical boundary conditions and the layer thickness. A proper ZnO layer thickness can be selected that ensures a zero *K*^2^ of the SAW thus allowing a selective transduction of the Love mode for the STFM configuration. Otherwise, for ZnO layer thickness corresponding to a non-zero Rayleigh and Love mode *K*^2^, a lab-on-chip system suitable for microfluidics and biosensing applications can be implemented that benefits from the propagation of both the two waves. 

The available literature reports many experimental investigations of Love wave sensors that include a non-piezoelectric silicon dioxide or/and a polymer (PMMA) layer on a piezoelectric ST-cut quartz substrate [16,17,18]. A rough estimation of its highest mass sensitivity is 380 cm^2^/g for a SiO_2_ layer normalized thickness of 14%, while a maximum sensitivity of 430 cm^2^/g was achieved with PMMA guiding layer thickness of around 3.5% of wavelength [19,20]. In reference 17 SiO_2_ and PMMA guiding layers were combined and an experimental sensitivity as high as 510 cm^2^/g was obtained. 

The design of an enhanced performance Love wave sensor requires a proper choice of several parameters, such as the acoustic mode order, substrate and layer material types, layer thickness, and the electrical boundary conditions. Last but not least is the choice of the IDTs, i.e., single electrode or double electrode, for example. Sensors with single phase unidirectional transducer (SPUDT) or split electrode configuration are reported to have low noise and insertion loss, thus leading to better sensitivity to surface perturbation. However it should be noted that sensitivity is also a function of electrode type, number of fingers, width and pitch of finger, delay path length and substrate selection. Moreover, by choosing layer materials with temperature coefficients of delay with opposite signs, it is possible to design a thermally compensated sensor, or at least to reduce the temperature sensitivity of the acoustic mode velocity. 

In conclusion, improvement in sensitivity, linearity and stability is possible by proper design of the overall device that includes the thickness and material types of the guiding layer, substrate and even an additional layer to be positioned onto the piezoelectric layer.

## 8. Conclusions 

The theoretical sensitivity of Love wave sensors to the properties of a liquid environment and to a mass deposited from a viscous Newtonian liquid phase is derived. Numerical calculations were performed for the case of a glass substrate covered by a ZnO layer with its c-axis 30° tilted with respect to the substrate normal. The guiding layer was supposed to contact both the vacuum and a viscous liquid, and to be bare or covered by an interface mass layer in order to estimate the mass sensitivity of the sensor in liquid. The attenuation, phase and group velocity of the first Love mode propagating along the ZnO/glass-based structures have been theoretically investigated for different ZnO thicknesses, liquid mass density and viscosity. The calculation of the real and imaginary parts of the phase velocity allowed the estimation of both the phase and group velocity and propagation loss sensitivities to the liquid viscosity and density, and to a mass anchored to the ZnO surface, for different ZnO thicknesses. As a result, by adjusting the ZnO layer to an optimum thickness value, a sensitivity peak results in changes in the physical properties of the medium contacting its surface. Moreover, due to the different phase and group velocity sensitivities, the Love mode sensor exhibits three responses to the changed environmental parameters, the attenuation and the two velocity shifts. Due to the simultaneous propagation of both the Love and Rayleigh waves, a lab-on-chip structure can be designed for microfluidic and biosensing applications. 

## Figures and Tables

**Table 1:** The mode order, the ZnO normalized thickness, the gravimetric sensitivity in vacuum and the *K*^2^ of the STF configurations. The ZnO normalized thicknesses refer to the peak of the phase velocity dispersion derivative.

Mode	ZnO h/λ	S_m_·λ(·10^−6^)·m^3^·kg^−1^	*K*^2^ (%)
L_1_	0.047	−557	0.13
L_2_	0.65	−150	0.035
L_3_	1.25	−90	0.03
L_4_	1.85	−60	0.024
L_5_	2.45	−50	0.02
Rayleigh wave	0.044	−538	0.224

## Supplementary Materials

The following are available online at www.mdpi.com/2079-6374/6/4/59/s1, Figure S1: The half-space/guiding layer system, Figure S2: The half-space/guiding layer/viscous liquid system, Figure S3: The half-space/guiding layer/mass layer/viscous liquid system.
