# Supplementary material for: A Theoretical Study of Love Wave Sensors Based on ZnO–Glass Layered Structures for Application to Liquid Environments
Source: Biosensors (Basel). 2016 Dec 2;6(4):59. doi: 10.3390/bios6040059 (PMC5192379; doi:10.3390/bios6040059)
Supplement: Supplementary file 1 [file biosensors-06-00059-s001.pdf]

Supplementary Materials: A Theoretical Study of Love Wave Sensors Based on ZnO–Glass Layered Structures for Application to Liquid Environments

Cinzia Caliendo and Muhammad Hamidullah

1. Half-Space/Guiding Layer

A piezoelectric ZnO layer of thickness h_{gl} overlays a glass isotropic half-space, as shown in Figure S1. The space above the layer is occupied by air or vacuum which is assumed to have no mechanical contact with the layer.

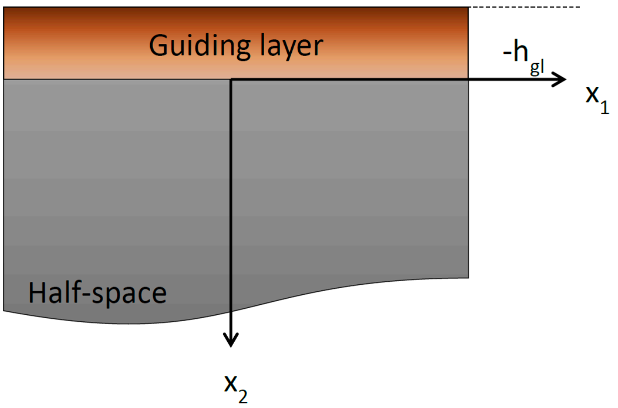

Figure S1: The half-space/guiding layer system.

The ZnO layer has its c-axis 30° tilted with respect to the surface normal. The ZnO stiffness constants $c_{\alpha\beta}$, piezoelectric constants $e_{\alpha\beta}$, and dielectric constants $\varepsilon_{\alpha\beta}$ were rotated by applying the Bond matrix method described in [1]. The coordinate system used through the paper is the following: the x_2 axis is parallel to the surface normal, the x_3 as axis is parallel to the wave polarization vector, and the x_1 axis is parallel to the wave propagation direction. In the quasi-static approximation, the matrix notation for the piezoelectric constitutive equations of the c-axis 30° tilted ZnO is the following:

$$\begin{pmatrix} T_1 \\ T_2 \\ T_3 \\ T_4 \\ T_5 \\ T_6 \\ D_1 \\ D_2 \\ D_3 \end{pmatrix} = \begin{pmatrix} c_{11} & c_{12} & c_{13} & c_{14} & 0 & 0 & 0 & -e_{31} & -e_{31} \\ c_{12} & c_{22} & c_{23} & c_{24} & 0 & 0 & 0 & -e_{31} & -e_{31} \\ c_{13} & c_{23} & c_{33} & c_{34} & 0 & 0 & 0 & -e_{31} & -e_{31} \\ c_{14} & c_{24} & c_{34} & c_{44} & 0 & 0 & 0 & -e_{31} & -e_{31} \\ 0 & 0 & 0 & 0 & c_{55} & c_{56} & -e_{31} & 0 & 0 \\ 0 & 0 & 0 & 0 & c_{56} & c_{66} & -e_{31} & 0 & 0 \\ 0 & 0 & 0 & 0 & e_{15} & e_{16} & \varepsilon_{11} & 0 & 0 \\ e_{21} & e_{22} & e_{23} & e_{24} & 0 & 0 & 0 & \varepsilon_{22} & \varepsilon_{23} \\ e_{31} & e_{32} & e_{33} & e_{34} & 0 & 0 & 0 & \varepsilon_{23} & \varepsilon_{33} \end{pmatrix} \cdot \begin{pmatrix} S_1 \\ S_2 \\ S_3 \\ S_4 \\ S_5 \\ S_6 \\ E_1 \\ E_2 \\ E_3 \end{pmatrix} \quad \text{(S1)}$$

The wave under consideration is assumed to travel in the x_1-direction along a surface whose normal is in the x_2 direction, and to be polarized parallel to the x_3 direction. The only non null particle displacement component is U_3, and both U_3 and the electric potential Φ are independent of the x_3 coordinate: since travelling wave solutions are in the form $U_3 = U_3(x_1, x_2, t)$ and $\Phi = \Phi(x_1, x_2, t)$, then the Equation (S1) can be rewritten as

$$\begin{pmatrix} T_1 \\ T_2 \\ T_3 \\ T_4 \\ T_5 \\ T_6 \\ D_1 \\ D_2 \\ D_3 \end{pmatrix} = \begin{pmatrix} c_{11} & c_{12} & c_{13} & c_{14} & 0 & 0 & 0 & -e_{31} & -e_{31} \\ c_{12} & c_{22} & c_{23} & c_{24} & 0 & 0 & 0 & -e_{31} & -e_{31} \\ c_{13} & c_{23} & c_{33} & c_{34} & 0 & 0 & 0 & -e_{31} & -e_{31} \\ c_{14} & c_{24} & c_{34} & c_{44} & 0 & 0 & 0 & -e_{31} & -e_{31} \\ 0 & 0 & 0 & 0 & c_{55} & c_{56} & -e_{31} & 0 & 0 \\ 0 & 0 & 0 & 0 & c_{56} & c_{66} & -e_{31} & 0 & 0 \\ 0 & 0 & 0 & 0 & e_{15} & e_{16} & \varepsilon_{11} & 0 & 0 \\ e_{21} & e_{22} & e_{23} & e_{24} & 0 & 0 & 0 & \varepsilon_{22} & \varepsilon_{23} \\ e_{31} & e_{32} & e_{33} & e_{34} & 0 & 0 & 0 & \varepsilon_{23} & \varepsilon_{33} \end{pmatrix} \cdot \begin{pmatrix} 0 \\ 0 \\ 0 \\ 0 \\ S_5 \\ S_6 \\ E_1 \\ E_2 \\ 0 \end{pmatrix} \quad \text{(S2)}$$

The equations of motion for the piezoelectric finite thickness layer and for the isotropic half-space are:

$$\left.\begin{matrix} \rho_{gl}\dfrac{\partial^2 u_3}{\partial t^2} = \dfrac{\partial T_{i3}}{\partial x_i} \\ \dfrac{\partial D_i}{\partial x_i} = 0 \end{matrix}\right],\ 0 < x_2 < -h \quad \text{(S3)}$$

$$\left.\begin{matrix} \rho_{sub}\dfrac{\partial^2 u_3^{sub}}{\partial t^2} = c_{44}^{sub}\left(\dfrac{\partial^2}{\partial x_1^2} + \dfrac{\partial^2}{\partial x_2^2}\right)u_3^{sub} \\ \varepsilon_{11}^{sub}\left(\dfrac{\partial^2}{\partial x_1^2} + \dfrac{\partial^2}{\partial x_2^2}\right)\Phi = 0 \end{matrix}\right],\ x_2 > 0 \quad \text{(S4)}$$

$$\left(\frac{\partial^2}{\partial x_1^2} + \frac{\partial^2}{\partial x_2^2}\right)\Phi' = 0,\ x_2 < 0 \quad \text{(S5)}$$

The assumed solutions to the propagation equations in the substrate (sub), guiding layer (gl) and in the region above the free surface of the guiding layer are the following:

$$\left.\begin{matrix} u_3^s = [Ae^{-qx_2}] \cdot e^{j(kx_1 - \omega t)} \\ \Phi_{sub} = Be^{-qx_2}e^{j(kx_1 - \omega t)} \end{matrix}\right] \quad \text{(S6)}$$

$$\left.\begin{matrix} u_3^{gl} = [C_1 \cdot e^{jk\beta x_2} + C_2 \cdot e^{-jk\beta x_2}] \cdot e^{j(kx_1 - \omega t)} \\ \Phi_{gl} = [C_3 \cdot e^{jk\beta x_2} + C_4 e^{-jk\beta x_2}] \cdot e^{j(kx_1 - \omega t)} \end{matrix}\right] \quad \text{(S7)}$$

$$\Phi_{air} = \Phi_{gl}(x_2 = 0)e^{k(x_2 + h_{gl})}] \quad \text{(S8)}$$

where C_1, C_2, C_3, C_4, A and B are arbitrary constants, k=ω/v is the wave-number (it is real since the ZnO and glass are lossless materials), v is the Love mode velocity (whose value is in between the shear horizontal bulk acoustic wave velocity in the layer and in the substrate, v_{SH}^{sub} and v_{SH}^{gl}, $\omega = 2\pi f$, $f = v/\Lambda$, q and β account the variation in depth of the wave amplitude. By substituting Equations (S6–S8) into Equations (S3–S5), two system of equations for the displacement and the potential are obtained, that involve the layer and substrate material parameters. An algebraic equation in β and one in q are obtained by solving the secular equations for the layer and for the substrate. From the two algebraic equations, only q and β values are retained that correspond to a wave displacement that decay to zero with depth below the $x_2 = 0$ plane, and that varies sinusoidally into the layer. By substituting the Equations (S6–S8) into the boundary and continuity conditions, a set of homogeneous equations for the C_1, C_2, C_3, C_4, A and B coefficients are obtained with v as the unknown. By setting the determinant of the coefficients equal to zero, real values of v are found for fixed layer thickness and wavelength λ. An optimized numerical procedure was used to find a real velocity value that drives the size of the determinant of the coefficients as close to zero as possible.

2. Half-Space/Guiding Layer/Liquid

The guiding layer, as well as the half-space, is assumed to be isotropic with the constant c_{44} numerically equal to the stiffened value calculated in the previous paragraph. A viscous non

conductive liquid half-space contacts the upper surface of the layer, as shown in Figure S2: ρ_l and η are the liquid mass density and viscosity.

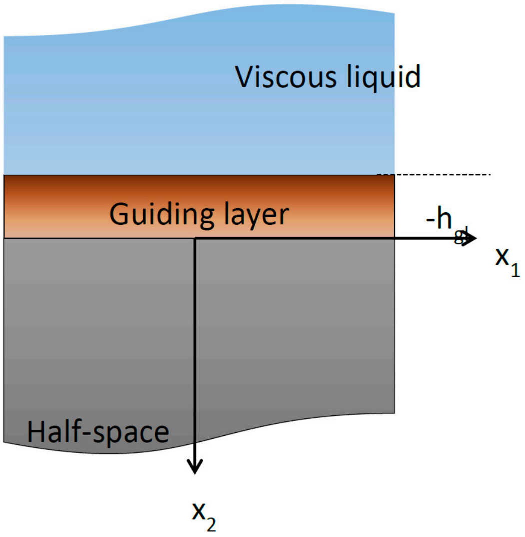

Figure S2. The half-space/guiding layer/viscous liquid system.

The equations of motion for the three media are the following:

$$\rho_{sub}\frac{\partial^2 u_3^{sub}}{\partial t^2} = c_{44}^{sub}\left(\frac{\partial^2}{\partial x_1^2}+\frac{\partial^2}{\partial x_2^2}\right)u_3^{sub},\ x_2 > 0 \quad \text{(S9)}$$

$$\rho_{gl}\frac{\partial^2 u_3^{gl}}{\partial t^2} = c_{44}^{gl}\left(\frac{\partial^2}{\partial x_1^2}+\frac{\partial^2}{\partial x_2^2}\right)u_3^{gl},\ 0 < x_2 < -h_{gl} \quad \text{(S10)}$$

$$\frac{\partial v_3}{\partial t} = \frac{\eta}{\rho_l}\left(\frac{\partial^2}{\partial x_1^2}+\frac{\partial^2}{\partial x_2^2}\right)v_3,\ x_2 < -h_{gl} \quad \text{(S11)}$$

The assumed solutions to the Equations (S9–S11) are the following:

$$u_3^s = [U_{sub}^0 \cdot e^{-qx_2}] \cdot e^{j(kx_1-\omega t)} \quad \text{(S12)}$$

$$u_3^{gl} = [U_{gl}^1 \cdot sin(bx_2) + U_{gl}^2 \cos(bx_2)] \cdot e^{j(kx_1-\omega t)} \quad \text{(S13)}$$

$$u_3^{liquid} = [U_{liq}^1 \cdot e^{\lambda_1 x_2}] \cdot e^{j(kx_1-\omega t)} \quad \text{(S14)}$$

where $k = k_0 + j \cdot \alpha$, $q^2 = k^2 - k_s^2$, $b^2 = k_{gl}^2 - k^2$, $k_s = \omega / v_{SHBAW}^s$, $k_{gl} = \omega / v_{SHBAW}^{gl}$, $v_{SHBAW}^s = \sqrt{c_{44}^s / \rho_s}$, $v_{SHBAW}^{gl} = \sqrt{c_{44}^{gl} / \rho_{gl}}$ and $\lambda_1^2 = k^2 - j\omega(\rho_l / \eta)$. The particle displacement and the traction components of the stress must be continuous across the $x_2 = 0$ and $x_2 = -h_{gl}$ interfaces. When the Equations (S12–S14) are substituted into the boundary conditions, a set of four homogeneous algebraic equations are obtained in the four coefficients U_{sub}^0, U_{gl}^1, U_{gl}^2, and U_{liq}^1 : a non trivial solution of this equations system exists if the determinant of the coefficients vanishes. The determinant is:

$$\begin{vmatrix} 0 & e^{-\lambda_1 h_{gl}} & -j\omega \cdot \sin(bh_{gl}) & j\omega \cdot \cos(bh_{gl}) \\ 0 & \eta \cdot \lambda_1 e^{-\lambda_1 h_{gl}} & -c_{44}^{gl} b \cdot \cos(bh_{gl}) & -c_{44}^{gl} b \cdot \sin(bh_{gl}) \\ 1 & 0 & 0 & -1 \\ -q \cdot c_{44}^{sub} & 0 & -c_{44}^{gl} \cdot b & 0 \end{vmatrix} = 0 \quad \text{(S15)}$$

From Equation (S15) the wave dispersion equation is obtained: the system of two equations, the real and imaginary parts of the dispersion equation, were numerically solved by using the Levenberg-Marquardt-Fletcher method implemented within a Matlab routine, and the real and imaginary parts of the Love wave velocity were then calculated, v^{im} and v^{real}.

3. Half-Space/Guiding Layer/Mass Layer/Liquid

An added mass layer (am) of thickness h_{am} is supposed to cover the guiding layer surface as shown in Figure S3. The guiding layer, the mass layer and the half-space are assumed to be isotropic.

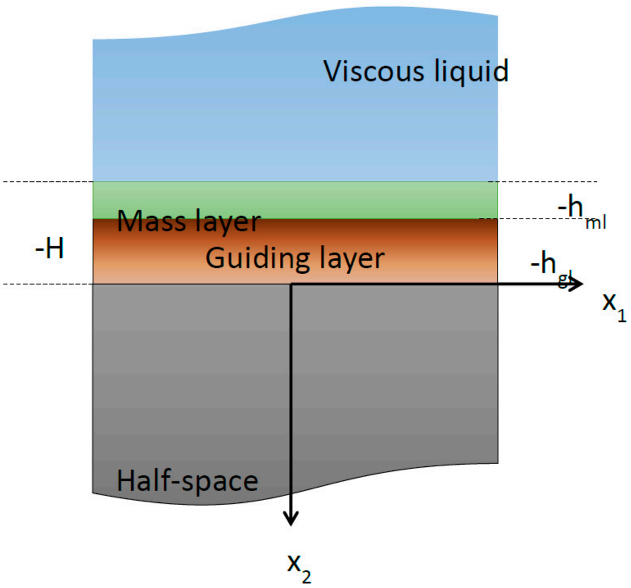

Figure S3. The half-space/guiding layer/mass layer/viscous liquid system.

The equations of motion for the four media are the following:

$$\rho_{sub} \frac{\partial^2 u_3^{sub}}{\partial t^2} = c_{44}^{sub} \left(\frac{\partial^2}{\partial x_1^2} + \frac{\partial^2}{\partial x_2^2} \right) u_3^{sub}, \ x_2 > 0 \quad \text{(S16)}$$

$$\rho_{gl} \frac{\partial^2 u_3^{gl}}{\partial t^2} = \overline{c_{44}^{gl}} \left(\frac{\partial^2}{\partial x_1^2} + \frac{\partial^2}{\partial x_2^2} \right) u_3^{gl}, \ 0 < x_2 < -h_{gl} \quad \text{(S17)}$$

$$\rho_{am} \frac{\partial^2 u_3^{am}}{\partial t^2} = c_{44}^{am} \left(\frac{\partial^2}{\partial x_1^2} + \frac{\partial^2}{\partial x_2^2} \right) u_3^{am}, \ -h_{gl} < x_2 < -h_{am} \quad \text{(S18)}$$

$$\frac{\partial v_3}{\partial t} = \frac{\eta}{\rho_l} \left(\frac{\partial^2}{\partial x_1^2} + \frac{\partial^2}{\partial x_2^2} \right) v_3, \text{ for } x_2 < -H \quad \text{(S19)}$$

The assumed solutions to the Equations (S16–S19) are the following:

$$u_3^{substrate} = [U_{sub}^0 \cdot e^{-qx_2}] \cdot e^{j(kx_1 - \omega t)} \quad \text{(S20)}$$

$$u_3^{gl} = [U_{gl}^1 \cdot cos(bx_2) + U_{gl}^2 \cdot \sin(bx_2)] \cdot e^{j(kx_1 - \omega t)} \tag{S21}$$

$$u_3^{am} = [U_{am}^1 \cdot cos(px_2) + U_{am}^2 \cdot \sin(px_2)] \cdot e^{j(kx_1 - \omega t)} \tag{S22}$$

$$u_3^{liquid} = [U_{liq}^1 \cdot e^{\lambda_1 x_2}] \cdot e^{j(kx_1 - \omega t)} \tag{S23}$$

The particle displacement and the traction components of stress must be continuous across the substrate/guiding layer, guiding layer/mass layer, and mass layer/liquid interfaces. When the Equation (S20–S23) are substituted into the boundary and continuity conditions, a set of six homogeneous algebraic equations are obtained in the six coefficients U_{sub}^0, U_{gl}^1, U_{gl}^2, U_{am}^1, U_{am}^2, and U_{liq}^1: a non trivial solution of this equations system exists if the determinant of the coefficients vanishes. The determinant is:

$$\begin{vmatrix} 1 & -1 & 0 & 0 & 0 & 0 \\ -qc_{44}^{sub} & 0 & -bc_{44}^{gl} & 0 & 0 & 0 \\ 0 & \cos(bh_{gl}) & -\sin(bh_{gl}) & -\cos(ph_{gl}) & \sin(ph_{gl}) & 0 \\ 0 & c_{44}^{gl} bsin(bh_{gl}) & c_{44}^{gl} bcos(bh_{gl}) & -c_{44}^{ml} psin(ph_{gl}) & -c_{44}^{ml} pcos(ph_{gl}) & 0 \\ 0 & 0 & 0 & -j\omega\cos(pH) & j\omega\sin(pH) & -e^{-\lambda_1 H} \\ 0 & 0 & 0 & c_{44}^{ml} psin(pH) & c_{44}^{ml} pcos(pH) & -\eta\lambda_1 e^{-\lambda_1 H} \end{vmatrix} = 0 \tag{S24}$$

where $q^2 = k^2 - k_s^2$, $b^2 = k_{gl}^2 - k^2$, $p^2 = k_{am}^2 - k^2$, $\lambda_1^2 = k^2 - j\omega(\rho_l / \eta)$, $k_{am} = {}^{\omega}/_{v_{SHBAW}^{am}}$, and H = h_{gl} + h_{am}. From Equation (S24) the wave dispersion equation is obtained: the system of two equations, the real and imaginary parts of the dispersion equation, were numerically solved by using the Levenberg-Marquardt-Fletcher method implemented within a Matlab routine, and the real and imaginary parts of the Love wave velocity were then calculated, v^{im} and v^{real}.

Reference

1. Auld, B.A. *Acoustic Fields and Waves in Solids*; 2nd ed., R.E. Krieger Publishing: Malabar, FL, USA, 1990; Volume 1.
